# Barriers to Disclosure of Disability and Request for Accommodations Among First-Year Resident Physicians in the US

**DOI:** 10.1001/jamanetworkopen.2023.9981

**Published:** 2023-05-11

**Authors:** Karina Pereira-Lima, Lisa M. Meeks, Katherine E. T. Ross, Jasmine R. Marcelin, Lydia Smeltz, Elena Frank, Srijan Sen

**Affiliations:** 1 Department of Neurology, University of Michigan Medical School, Ann Arbor; 2 Department of Learning Health Sciences, University of Michigan Medical School, Ann Arbor; 3 Department of Family Medicine, University of Michigan Medical School, Ann Arbor; 4 Department of Psychology, University of Michigan, Ann Arbor; 5 Department of Internal Medicine, University of Nebraska Medical School, Omaha; 6 currently a medical student at Penn State College of Medicine, Hershey, Pennsylvania; 7 Michigan Neuroscience Institute, University of Michigan Medical School, Ann Arbor; 8 Eisenberg Family Depression Center, University of Michigan Medical School, Ann Arbor

## Abstract

**Importance:**

Ensuring access to accommodations is critical for resident physicians and their patients. Studies show that a large proportion of medical trainees with disabilities do not request needed accommodations; however, drivers of nonrequests are unknown.

**Objective:**

To assess the frequency of accommodation requests among first-year resident physicians (ie, interns) with disabilities and to identify possible drivers of nonrequest for needed accommodations.

**Design, Setting, and Participants:**

As part of the Intern Health Study, a longitudinal cohort study of first-year resident physicians, residents at 86 surgical and nonsurgical residency programs in 64 US institutions provided demographic and training characteristics 2 months prior to matriculation (April-May 2021). At the end of their intern year (June 2022), participants completed a new survey with questions about disability-related information, including disability status, disability type, whether they received accommodations, and if not, reasons for nonaccommodation. Poststratification and attrition weights were used to estimate the frequency of accommodation requests and reasons for not requesting accommodations. Interns reporting at least 1 disability were included in the analysis.

**Main Outcomes and Measures:**

Prevalence of reported disabilities, residency specialties distribution, frequency of accommodation requests, and reasons for nonaccommodation among resident physicians with disabilities.

**Results:**

Among the 1486 resident physicians who completed the baseline survey, 799 (53.8%) replied to the disability questions. Of those, 94 interns (11.8%; weighted number, 173 [11.9%]) reported at least 1 disability and were included in the present study (weighted numbers, 91 [52.6%] men, 82 [47.4%] women, mean [SD] age, 28.6 [3.0] years). Among interns with reported disability and need for accommodations (83 of 173 [48.0%]), more than half (42 [50.6%]) did not request them. The most frequently reported reasons for not requesting needed accommodations were fear of stigma or bias (25 [59.5%]), lack of a clear institutional process for requesting accommodations (10 [23.8%]), and lack of documentation (5 [11.9%]).

**Conclusions and Relevance:**

Program directors should investigate cultural and structural factors within their programs that contribute to an environment where residents do not feel safe or supported in disclosing disability and requesting accommodation and review their disability policies for clarity.

## Key Points

**Question:**

Why do resident physicians with disabilities refrain from requesting needed accommodations?

**Findings:**

In this cohort study including a weighted sample of 173 first-year resident physicians with disabilities, 50.6% of those with reported need for accommodations did not request them. Fear of stigma or bias and lack of a clear institutional process were the most reported reasons for not requesting needed accommodations.

**Meaning:**

These findings suggest that greater transparency and compliance with best policies for disability disclosure systems are needed in graduate medical education, and program directors should investigate factors contributing to an unsafe or unsupported environment for requesting accommodation within their programs.

## Teaser

This cohort study assesses the frequency of disability reporting and request for accommodations among first-year resident physicians with disabilities and identifies possible drivers of nonrequest for needed accommodations among this population.

## Introduction

The addition of new disability-focused regulations by the Accreditation Council for Graduate Medical Education (ACGME)^1^ demonstrates an increasing commitment to disability as a key component of diversity, equity, and inclusion efforts in medical training. However, compliance with requirements and recommendations remains low.^2^ Furthermore, qualitative studies^3,4,5,6^ and anecdotal reports^7,8,9^ suggest that stigmatizing attitudes toward disability and an embedded culture of ableism in medicine are barriers frequently encountered by medical trainees with disabilities.

Disability disclosure and access to accommodations have significant implications for trainees with disabilities and their patients.^10^ In fact, recent studies highlight the critical nature of program access, defined as access to accommodations or not needing accommodations due to an environment where access needs are already met,^10,11^ to the well-being and performance of medical trainees with disabilities. Specifically, in a 2021 study,^10^ researchers found that program access was associated with a lower increase in depressive symptoms, as well as with a lower frequency of self-reported medical errors among resident physicians with disabilities. In the following year, studies focused on medical students with disabilities found that program access was associated with better well-being^11^ and academic success^12^ in this population.

While prior studies demonstrate that a large proportion of medical trainees do not request accommodations when needed,^13^ little is known about drivers of nonrequests. Herein, using data from the University of Michigan Intern Health Study, a longitudinal cohort study of first-year resident physicians, we aimed to assess the frequency of disability reporting and request for accommodations among resident physicians with disabilities and to identify possible drivers of nonrequest for disability accommodations in this population.

## Methods

The University of Michigan Institutional Review Board approved this study. All participants provided informed consent electronically and received between $80 to $130 in compensation. This study follows the Strengthening the Reporting of Observational Studies in Epidemiology (STROBE) reporting guideline.

The Intern Health Study is a longitudinal cohort study that assesses the role of psychological, biological, and environmental factors in the development of depression under stress.^14^ Following the 2021-2022 academic year national residency match in March 2021, email addresses for incoming first-year residents across all specialties throughout the US were gathered from residency programs and publicly available databases. As part of this larger study, 4611 eligible intern physicians (ie, incoming first-year resident physicians in US residency programs offering graduate year 1 positions available immediately after completion of medical school) were invited via email to complete a web-based baseline confidential survey 2 months before beginning their first year of residency (ie, intern year). This baseline survey included questions about their demographic characteristics (sex, age, sexual orientation, and race and ethnicity, important to assign appropriate sample weights based on the demographic distribution of the entire population of intern physicians in the US), specialty, and residency institution. At the end of their intern year (June 2022), participants completed a survey with disability-related questions that directly mirrored those in the Association of American Medical Colleges (AAMC) year 2 and graduation questionnaires, assessing whether a resident self-identifies as a person with a disability (yes, no, or I don’t know), type of disability (attention-deficit/hyperactivity disorder, chronic health disability, deaf or hard of hearing, learning disability, mobility disability, psychological disability, visual disability, or other), and whether the program has provided disability accommodations (yes or no). We also included the question about why residents did not receive disability accommodations with additional response choices to allow participants to provide more information about possible drivers of nonrequests, including stigma, bias, unclear institutional policies and procedures, and lack of documentation (survey questions are available in eMethods in Supplement 1). In keeping with prior studies,^13^ interns who indicated they requested accommodations (ie, those who requested and had accommodations provided or denied or those whose request was under review) and those who indicated they did not request accommodations for reasons other than not needing accommodations were coded as needing accommodation.

Participants from the parent study who replied “yes” or “I don’t know” to the disability status question were presented with follow-up questions on disability type and accommodations. Among those, all interns who reported at least 1 type of disability (ie, attention-deficit/hyperactivity disorder, chronic health, deaf or hard of hearing, learning, mobility, psychological, visual, or other type) were eligible for inclusion in the present study. The decision of including all interns who reported at least 1 type of disability was based on the right of resident physicians with any type of disability to reasonable, needed accommodations.

### Survey Weights Strategy

In accordance to previously described methods,^15^ poststratification^16^ and attrition^17^ weights were used to reduce potential bias due to nonrepresentative sampling and to account for the differences between participants who completed and those who did not complete the follow-up survey. For the poststratification weights,^16^ the demographic composition (ie, sex, race and ethnicity, and surgical or nonsurgical specialty) of the complete set of US intern physicians in 2021 according to population data obtained from the AAMC was used as the target population. The AAMC data set included demographic information on the overall population of US intern physicians and by residency specialty. We coded individual residency specialties as surgical and nonsurgical using the American College of Surgeons classification.^18^ Within each specialty group (ie, surgical vs nonsurgical), we obtained the number of women and men and the numbers of underrepresented minority and Asian and White (ie, not underrepresented) first-year residents. We then used the R package anesrake^16^ to generate poststratification weights in 2 steps: first generating weights by specialty group within the 2021 cohort (ie, surgical vs nonsurgical) as the ranking variable (w1a), and then generating weights with sex and race and ethnicity within each specialty group as the ranking variable (w1b), so that the application of those weights on the present study sample would result in a sample with a distribution of specialty, sex, and race and ethnicity that matches the distribution of the AAMC populational data.

For the attrition weights, we first performed a least absolute shrinkage and selection operator (LASSO) regression to identify which baseline factors significantly predicted completion of the disability questions at month 12 of internship among interns who enrolled in the baseline survey. Two variables were identified and therefore selected to be included in the estimation of attrition weights: sex assigned at birth (men vs women) and specialty group (surgical vs nonsurgical). We then used the R package twang^17,19^ to estimate the propensity score of disability status question response (p). This score was calculated using the default settings of the R function ps, which uses linear regression gradient boosting from the gbm package. Other default settings can be found in the twang package documentation.^19^ The resulting propensity score can be understood as the probability of treatment assignment (in this case, response to the disability status question) given a set of covariates (in this case, the set of covariates previously identified with the LASSO regression). Subsequently, we extracted attrition weights (w2a) from the propensity score using the R function get.weights. The attrition weight for each participant whom we included in the analysis is 1 divided by p, where p is the propensity score.

After obtaining poststratification and attrition weights using these procedures, we calculated the total weights as w1a × w1b × w2a. The svdesign function of the R package survey^20,21^ was used to incorporate the generated survey weights into the present study data set.

A 2-sided *P* < .05 was considered statistically significant for all statistical analyses. Analyses were conducted in R, version 4.2.2 (R Program for Statistical Computing).

## Results

Among the 1486 incoming intern physicians who enrolled in the parent study (32.2%), 799 (53.8%) completed the end-of-year (June 2022) follow-up survey containing the disability questions (323 [40.4%] men, 438 [54.8%] women, and 38 [4.8%] missing sex; mean [SD] age, 27.7 [2.8] years). A total of 74 residents (9.3%) responded “yes” to the disability status question, 27 (3.4%) replied “I don’t know,” and 698 (87.4%) replied “no.” Collectively, 94 intern physicians who replied “yes” or “I don’t know” to the disability status question (11.8%; weighted number, 173 [11.9%]) went on to report at least 1 type of disability and thus were included in the present study analysis (weighted numbers: 91 [52.6%] men and 82 [47.4%] women; mean [SD] age, 28.6 [3.0] years; 53 [30.6%] underrepresented in medicine [including African American, Arab or Middle Eastern, Hispanic or Latino, and multiple races] and 120 [69.4%] not underrepresented). Participants represented 86 residency programs across 15 surgical and nonsurgical specialties at 64 US institutions from all geographic census regions in the US (Table 1). The most reported disability types were attention-deficit/hyperactivity disorder (weighted number, 124 [71.7%]), chronic health (weighted number, 28 [16.2%]), psychological (weighted number, 14 [8.1%]), and deaf or hard of hearing (weighted number, 11 [6.4%]) disabilities (Table 2).

**Table 1.** Demographic and Training Characteristics of Participants Who Reported ≥1 Type of Disability^a^

Characteristic	Weighted sample (n = 173)^b^	Unweighted sample (n = 94)
**Demographic **			
Sex		
Men	91 (52.6)	40 (42.6)
Women	82 (47.4)	49 (52.1)
Missing	0	5 (5.3)
Age, mean (SD), y	28.6 (3.0)	28.5 (2.9)
Sexual orientation		
Heterosexual	120 (69.4)	66 (70.2)
Lesbian, gay, bisexual, or other^c^	52 (30.1)	28 (29.8)
Race and ethnicity		
Underrepresented in medicine^d^	53 (30.6)	28 (29.8)
Not underrepresented in medicine	120 (69.4)	66 (70.2)
**Specialty information **			
Surgical specialties		
Obstetrics and gynecology	8 (4.6)	5 (5.3)
Ophthalmology	1 (0.6)	1 (1.1)
Surgery-general	11 (6.4)	7 (7.4)
Nonsurgical specialties		
Anesthesiology	11 (6.4)	5 (5.3)
Child neurology	0	1 (1.1)
Emergency medicine	6 (3.5)	3 (3.2)
Family medicine	18 (10.4)	10 (10.6)
Internal medicine	58 (33.5)	29 (30.9)
Internal medicine/emergency medicine	2 (1.2)	1 (1.1)
Neurology	5 (2.9)	3 (3.2)
Pediatrics	22 (12.7)	11 (11.7)
Physical medicine and rehabilitation	3 (1.7)	1 (1.1)
Psychiatry	23 (13.3)	14 (14.9)
Radiology-diagnostic	4 (2.3)	2 (2.1)
Transitional year	2 (1.2)	1 (1.1)
**Geographic census region**			
Midwest	32 (18.5)	19 (20.2)
Northeast	46 (26.6)	26 (27.7)
South	70 (40.5)	36 (38.3)
West	25 (14.5)	13 (13.8)

^a^ Unless otherwise indicated, data are expressed as No. (%) of residents.

^b^ Weighted numbers were rounded to the nearest integer; percentages were calculated using rounded totals. Weighted totals may sum to more than weighted numbers due to rounding.

^c^ Includes interns who selected “gay/lesbian,” “bisexual,” or “other___” in the multiple-choice survey question about sexual orientation (eMethods in Supplement 1). Open-text responses to the category other included “queer” and “unknown.”

^d^ Residents were coded as underrepresented in medicine based on the American Association of Medical Colleges definition as “racial and ethnic populations that are underrepresented in the medical profession relative to their numbers in the general population.” In this study sample, this group included first-year residents self-identifying as African American, Arab or Middle Eastern, Hispanic or Latino, and multiple races.

**Table 2.** Disability-Related Characteristics of Participants

Characteristic	No. (%)	
	Weighted sample (n = 173)^a^	Unweighted sample (n = 94)
Disability type^b^		
Attention-deficit/hyperactivity disorder	124 (71.7)	65 (69.1)
Chronic health disability	28 (16.2)	16 (17.0)
Deaf or hard of hearing	11 (6.4)	6 (6.4)
Learning disability	9 (5.2)	5 (5.3)
Mobility disability	3 (1.7)	2 (2.1)
Psychological disability	14 (8.1)	8 (8.5)
Visual disability	2 (1.2)	1 (1.1)
Other functional impairment	5 (2.9)	4 (4.3)
Did not request accommodations because does not need accommodations (no)	81 (46.8)	44 (46.8)
Need for accommodation (yes)^c^		
Requested accommodations: accommodation was provided by the medical school	40 (23.1)	19 (20.2)
Requested accommodations: accommodation request was under review by residency program	2 (1.2)	1 (1.1)
Requested accommodation: accommodation request was denied by residency program	0	0
Did not request accommodation due to fear of stigma or bias	25 (14.5)	11 (11.7)
Did not request accommodation due to the lack of a clear institutional process to request accommodation	10 (5.8)	5 (5.3)
Did not request accommodation due to the lack of documentation to support the request	5 (2.9)	3 (3.2)
Did not request accommodations for other reasons	8 (4.6)	7 (7.4)
Need for accommodation missing	9 (5.2)	5 (5.3)

^a^ Weighted numbers were rounded to the nearest integer; percentages were calculated using rounded totals. Weighted totals may sum to more than weighted numbers due to rounding.

^b^ Percentages may not sum to 100% due to the possibility of interns reporting multiple types of disability.

^c^ Percentages may not sum to 100% due to the possibility of interns reporting multiple reasons to not request accommodations.

Approximately half of residents with disabilities reported needing accommodations (weighted number, 83 [48.0%]). Of those, most did not request them (weighted number, 42 [50.6%]). When asked about reasons for nonaccommodation, most residents who did not request needed accommodations reported fear of stigma or bias (weighted number, 25 [59.5%]), followed by lack of a clear institutional process for requesting accommodations (weighted number, 10 [23.8%]), and lack of documentation (weighted number, 5 [11.9%]). Most residents who reported requesting accommodations (weighted number, 40 [97.6%]) received them (Table 3).

**Table 3.** Accommodation Requests Among Intern Physicians With Reported Need for Accommodations

Request status/reason for not requesting	No. (%)					
	Weighted sample^a^			Unweighted sample		
	Requested accommodations (n = 41)	Did not request accommodations (n = 42)	Total needing accommodations (n = 83)	Requested accommodations (n = 20)	Did not request accommodations (n = 25)	Total needing accommodations (n = 45)
Requested accommodations						
Accommodation was provided	40 (97.6)	NA	40 (48.2)	19 (95.0)	NA	19 (42.2)
Request is under review	2 (4.9)	NA	2 (2.4)	1 (5.0)	NA	1 (2.2)
Request was denied	0	NA	0	0	NA	0
Did not request accommodations						
Due to fear of stigma or bias	NA	25 (59.5)	25 (30.1)	NA	13 (52.0)	13 (28.9)
Due to institution not having a clear process for requesting accommodations	NA	10 (23.8)	10 (12.0)	NA	6 (24.0)	6 (13.3)
Due to lack of documentation to support accommodation request	NA	5 (11.9)	5 (6.0)	NA	3 (12.0)	3 (6.7)
Due to other reasons^b^	NA	8 (19.0)	8 (9.6)	NA	7 (28.0)	7 (15.6)

Percentages may sum to more than 100% due to interns disclosing multiple reasons to not request accommodations.

^a^ Weighted numbers were rounded to the nearest integer; percentages were calculated using rounded totals. Weighted totals may sum to more than weighted numbers due to rounding.

^b^ Open-text responses to this category included: “not knowing what accommodations would look like” (n = 1), “wanting to figure out on my own” (n = 1), and “I did not tell my program I have ADHD [attention-deficit/hyperactivity disorder]” (N = 1).

## Discussion

In a 2021 survey, 9.3% of first-year residents across 15 surgical and nonsurgical specialties responded “yes” when asked if they were a person with a disability, representing a 24% increase in disability representation from the prior year.^10^ This growth is compatible with national trends observed among medical students.^22^ When asked about specific disabilities, a higher proportion (11.8%) of residents endorsed at least 1 type of disability. More than half of the residents needing disability accommodations (50.6%) did not request them. Fear of stigma or bias was the most prevalent reason for not requesting needed accommodations, followed by a lack of a clear institutional process for requesting accommodations. Once requested, most residents received accommodations.

These findings show a continued increase in disability representation and underscore the need for clear disability policies in residency. Given that approximately 3 in 5 residents who did not request needed accommodations reported fear of stigma or bias, programs should concentrate efforts on fostering an environment that supports psychological safety and where lived experiences of disability are viewed as valuable forms of diversity that enrich patient care. Indeed, a growing body of research suggests that diversity positively contributes to better patient care.^23,24^ This is particularly important in the context of disability, given the high rates of health care disparities encountered by disabled patients worldwide^25^ and studies showing that physicians frequently report concerns about their ability to provide quality care to patients with disabilities.^26,27,28,29^ Greater inclusion and support of residents with disabilities can challenge disability biases and stereotypes in medicine, leading to positive downstream effects on patient care and increasing understanding of disability by physician peers.^30^

Approximately 1 in 5 first-year residents who did not request needed accommodations reported a lack of a clear institutional process as a reason for nonrequest. This is particularly relevant given recent studies demonstrating poor national institutional compliance with ACGME requirements for maintaining a disability policy.^2^ Taken together, these findings underscore the urgent need for programs to commit to ACGME compliance and design policies that include clear processes for requesting accommodations.

### Limitations

This study has some limitations. First, the Intern Health Study only captures data from first-year resident physicians. Importantly, while generalizations to other residency years should be made with caution, this group is the most proximal to requesting accommodations for the remainder of their residency program. Second, while our results suggest that most residents who reported requesting accommodations received them, we did not assess the type or quality of accommodations received nor the disability climate in which they were delivered. Third, our assessment on the need and request for accommodations was based on the categorization of survey responses on accommodation provision and reasons for lack of accommodations, rather than direct affirmations about nonrequests for accommodations when needed. Fourth, while poststratification and attrition survey weights were used to reduce bias due to nonrepresentative sampling and survey attrition, residents who did not participate may have different reasons for not requesting accommodations. Fifth, the small sample size precluded us from performing multivariable analyses considering other factors, such as intersectional identity, that may contribute to nonrequests for needed disability accommodations. Last, although questions that mirror the AAMC surveys for students and physicians were chosen to allow for direct comparison, questions about disability may be interpreted differently by different trainees due to a number of cultural, personal, and practical aspects.

## Conclusions

To our knowledge, this cohort study is the first to systematically investigate possible drivers of nonrequest for accommodations, finding that most intern physicians who require accommodations do not request them due to fear of bias and lack of a clear institutional process to request accommodations. In addition, our findings show that when accommodations were requested in this sample, most were approved. While this is promising, our study did not allow for a review of the quality of such accommodations and whether all accommodations necessary to reduce program barriers were provided. Future studies should investigate whether accommodated resident physicians are satisfied with the quality and efficacy of accommodations. Moreover, future research is needed on the types of accommodations requested and received, as well as on which types of accommodations were not requested when needed.

Given the large proportion of resident physicians not requesting needed accommodations, and the known negative outcomes associated with lack of program access among medical trainees with disabilities,^10,11,12^ program directors should investigate cultural and structural factors within their programs that contribute to an unsafe or unsupported environment for disclosing disability and requesting accommodation. Furthermore, residency programs have a responsibility to acknowledge disability as diversity and commit to evolving the culture of graduate medical education training through a lens of equity, such that residents with disabilities can train in inclusive and accessible programs.

## Notes

Supplement 1. **eMethods.** Survey Questions Used in the Present Study

Supplement 2.
Data Sharing Statement
