# Supplementary material for: Barriers to Disclosure of Disability and Request for Accommodations Among First-Year Resident Physicians in the US
Source: JAMA Netw Open. 2023 May 11;6(5):e239981. doi: 10.1001/jamanetworkopen.2023.9981 (PMC10176117; doi:10.1001/jamanetworkopen.2023.9981)
Supplement: Supplement 1. — eMethods. Survey Questions Used in the Present Study [file jamanetwopen-e239981-s001.pdf]

Supplementary Online Content

Pereira-Lima K, Meeks LM, Ross KET, et al. Barriers to disclosure of disability and request for accommodations among first-year resident physicians in the US. *JAMA Netw Open*. 2023;6(5):e239981. doi:10.1001/jamanetworkopen.2023.9981

eMethods. Survey Questions Used in the Present Study

This supplementary material has been provided by the authors to give readers additional information about their work.

© 2023 Pereira-Lima K et al. *JAMA Network Open*.

eMethods. Survey Questions Used in the Present Study

Demographic questions

• Date of Birth
(MM/DD/YYYY)

• Sex assigned at birth
( ) Male
( ) Female

• Which of the following best describes your sexual orientation?
( ) Heterosexual
( ) Gay/Lesbian
( ) Bisexual
( ) Prefer not to say
( ) Other _______________

• Ethnicity (check all that apply)
( ) White
( ) Black/African American
( ) Latino/Hispanic
( ) Asian (e.g. Indian, Chinese)
( ) Arab/Middle Eastern
( ) Native American
( ) Pacific Islander
( ) Other ________________

<u>End-of-year (12-month of internship) survey questions used in the present study</u>

Disability status

• Are you a person with a disability (e.g., ADHD, learning, psychological, chronic health, mobility, hearing, vision, etc.)?
( ) Yes
( ) No
( ) I do not know

Disability type

© 2023 Pereira-Lima K et al. *JAMA Network Open*.

• Which of the following best describes your disability? If you have more than one type, select all that apply.
( ) Attention deficit/hyperactivity disorder
( ) Chronic Health Disability
( ) Deaf or hard of hearing
( ) Learning disability
( ) Mobility disability
( ) Psychological disability
( ) Visual disability
( ) Other _______________

Accommodation provision

• Has your residency program provided accommodations for your disability?
( ) Yes
( ) No

Reason for non-accommodation

• Which of the following best describes why your residency program did not or has not provided accommodations:
( ) My request for accommodations was denied
( ) My request for accommodations is under review
( ) I have not requested accommodations because I feel I do not need accommodations
( ) Fear of stigma or bias
( ) I do not have documentation to support my request
( ) My institution does not have a clear process for requesting accommodation
( ) Other _______________

© 2023 Pereira-Lima K et al. *JAMA Network Open*.
