# Supplementary material for: Barriers to Disclosure of Disability and Request for Accommodations Among First-Year Resident Physicians in the US
Source: JAMA Netw Open. 2023 May 11;6(5):e239981. doi: 10.1001/jamanetworkopen.2023.9981 (PMC10176117; doi:10.1001/jamanetworkopen.2023.9981)
Supplement: Supplement 2. — Data Sharing Statement [file jamanetwopen-e239981-s002.pdf]

Data Sharing Statement

Pereira-Lima. Barriers to Disclosure of Disability and Request for Accommodations Among First-Year Resident Physicians in the US. *JAMA Netw Open*. Published May 11, 2023. doi:10.1001/jamanetworkopen.2023.9981

Data
Data available: Yes
Data types: Deidentified participant data
How to access data: meeksli@med.umich.edu
When available: With publication

Supporting Documents
Document types: Statistical/analytic code
How to access documents: meeksli@med.umich.edu
When available: With publication

Additional Information
Who can access the data: Researchers whose proposed use of the data has been approved and aligns with lab mission and vision.
Types of analyses: Specified purpose
Mechanisms of data availability: Data are identified through ICPSR: https://www.openicpsr.org/openicpsr/project/129225/version/V1/view
